# Resection of a juvenile nasopharyngeal angiofibroma via a hybrid cranioendoscopic approach

**DOI:** 10.3171/2020.4.FocusVid.19923

**Published:** 2020-04-01

**Authors:** Evan Joyce, Michael Karsy, Serge Makarenko, Jeramiah Alt, Richard Orlandi, William T. Couldwell

**Affiliations:** 1 Department of Neurosurgery, Clinical Neurosciences Center, University of Utah; and; 2 Division of Otolaryngology, University of Utah, Salt Lake City, Utah

**Keywords:** juvenile nasopharyngeal angiofibroma, JNA, microsurgical, endoscopic, cranioendoscopic, video

## Abstract

Endoscopic and open microsurgical approaches for pediatric patients are useful for a wide variety of skull base pathologies. A hybrid, cranioendoscopic approach may be beneficial in improving surgical resection for complex lesions. A case of a 13-year-old boy with a large juvenile nasopharyngeal angiofibroma extending through the nasopharynx and pterygopalatine fossa into the maxillary, sphenoid, and cavernous sinuses is demonstrated via an endoscopic, transnasal and frontotemporal, extended middle cranial fossa microsurgical approach. Management of a large pediatric tumor via narrow nasal passages, safe surgical resection around critical neurovascular structures, and complication avoidance is demonstrated.

The video can be found here: https://youtu.be/1WqvsOnQCxs.

## Transcript

### 0:25 Case presentation

This case is a 13-year-old boy who presented with epistaxis and nasal congestion. His MR imaging is degraded by artifact from his braces but it demonstrated a large skull base juvenile nasopharyngeal angiofibroma with extension and compression to the region of the inferior cavernous sinus and the horizontal carotid artery. These CT images show the real dimensions of the tumor and the extent of tumor in his face and nasal cavity.

### 0:58 Angiography and Embolization

Given the extensive vascularity of these lesions, angiography was performed, which demonstrated primarily blood supply from the left internal maxillary artery, demonstrated here. The catheter was placed, and PVA embolization ensued. Much of the vascularity was reduced by this maneuver. There was also some vascularity from the petrous segment of the carotid artery, which was not embolized.

### 1:25 Endoscopic resection

The facial part of the tumor will be removed transnasally, but we’ll access the oral cavity to retrieve tumor, as it is too large to remove through the nasal cavity. We’ll start with developing our mucosal margins around the anterior aspect of the tumor in the nasal cavity. The turbinate is cut, and the septum nasal mucosa is injected. We’ll then proceed with posterior septectomy. Now we’ll access the maxillary sinus on the left side and proceed with identification of the tumor through the posterior wall of the maxillary sinus. The tumor is identified, and we’ll start our perimeter dissection around the tumor, removing its attachment from the skull base. The maxillary sinus is widely opened, and we’ll proceed with dissection from the skull base and the infratemporal fossa region. The component within the pterygopalatine fossa is mobilized, and we’ll continue our circumferential dissection. After the remaining portion of the tumor is identified, we use a tissue debrider to allow full exposure of the attachment and then mobilize the most inferior and medial component. After the tumor is detached from the base of the skull, we mobilize the tumor down into the oropharynx. This is now retrieved through the oropharynx and removed. The remaining attachments to the bone are drilled to ensure complete removal. We purposefully left part of the tumor attached to the infratemporal fossa that is adjacent to the carotid artery. This will be dealt with in a combined fashion – both endoscopically from below and with control from above. The internal maxillary artery is then identified and ligated. Inspection of the parasellar area demonstrates no residual tumor in the midline.

### 4:21 Frontotemporal resection

We then proceed with our transcranial approach. We’ll perform a left frontotemporal craniotomy and a middle fossa floor dissection to dissect the tumor from the carotid artery and the 3rd division of the trigeminal nerve directly. We’ll identify the foramen spinosum and cut the middle meningeal artery. We open up the foramen rotundum and foramen ovale to provide us access to the region of the tumor. The 3rd division of the trigeminal nerve is dissected free of the roof of the tumor, and now we’ll dissect the tumor directly from the region of the horizontal segment of the carotid artery. Here we mobilize the tumor inferiorly, and we’ll drill out the canal of the carotid in the temporal bone. We’ll then proceed with removal of the tumor both from above and below. Having assured ourselves that we’ve removed all of the tumor, we’ll attain hemostasis, and this region will be inspected by our colleagues from below.

### 5:43 Closure

The craniotomy is closed with Medpor cranioplasty, and the scalp is closed.

### 5:50 Postoperative imaging

His postoperative imaging shows complete resection of the tumor. He was neurologically intact.

## Acknowledgments

We thank Vance Mortimer, our video editor, for his contribution to editing the operative videos.
